# Reference Values for Handgrip Strength in the Basque Country Elderly Population

**DOI:** 10.3390/biology9120414

**Published:** 2020-11-24

**Authors:** Xabier Río, Arkaitz Larrinaga-Undabarrena, Aitor Coca, Myriam Guerra-Balic

**Affiliations:** 1 Faculty of Psychology and Education, Physical Activity and Sports Sciences, University of Deusto, 48007 Bizkaia, Spain; a.larrinaga@deusto.es (A.L.-U.); aitor.coca@deusto.es (A.C.); 2 Faculty of Psychology, Education Sciences and Sport Blanquerna, University of Ramon Llul, 08022 Barcelona, Spain; miriamelisagb@blanquerna.url.edu

**Keywords:** handgrip strength, elderly, weakness, health-promoting, physical activity

## Abstract

**Simple Summary:**

Functional impairment is a growing global problem that increases with age and acute hospitalisations. Handgrip strength (HGS) is one of the tests used as a predictor of low skeletal muscle strength in the diagnosis of weakness. The aim of this study is to provide reference values of HGS in adults and older adults in the Basque Country by identifying cut-off points to measure weakness and compare the values with other populations. A health-promoting programme seems to be effective in obtaining better values as age increases with respect to the general population in the HGS test, delaying and even avoiding reaching the cut-off values for detecting weakness as a criterion for frailty. Despite the current findings available to health professionals for more effective detection of frailty, many of them have not been yet translated into clinical practice. Determining HGS values by population will allow to obtain clinically fast and effective cut-off values to detect weakness and probable risk in an ageing population.

**Abstract:**

Strength training is currently the most recommended primary therapeutic strategy to prevent and reverse the decline of muscle mass, strength, and functional deterioration associated with age. The aim is to provide reference values of handgrip strength (HGS) in the Basque Country population and compare the values with other populations. A total of 1869 subjects from the health-promoting programme for adults and older adults run by the Bilbao City Council were assessed using HGS with a digital dynamometer and anthropometric data measured by Tanita to obtain the mean values according to age distribution. From the 1869 subjects, 87.5% were women and 12.5% men. The HGS was higher among men than women, 32.4 ± 6.6 versus 20.1 ± 4.7 kg, respectively, *p* < 0.001 at all ages. Weak HGS cut-off points by age groups ranged from 31.0 to 23.8 and from 18.9 to 12.4 in men and women, respectively. The sample data were compared (*d*, t, and α) with those of other populations in all age groups (group > 60 years at 95% df, *p* < 0.05). A health-promoting programme appears to be effective in the general population in obtaining better values in the HGS test as age increases.

## 1. Introduction

Today’s multiple sedentary behaviours lead to an exponential increase in morbidity and mortality risk rates [1,2,3,4]. Cardiorespiratory capacity is known to be one of the most important factors in predicting mortality risk [2,5], but it has also been observed that greater muscle strength, as measured for example by handgrip strength (hereinafter, HGS), has been shown to reduce the risk of all-cause mortality [6,7]. Increased HGS has been associated with significant improvements in parameters such as high-density lipoprotein (HDL), triglycerides, systolic and diastolic blood pressure [8], in addition to correlating with quality of life [9]. On the other hand, low levels of HGS are associated with dementia [10], an important pathology as we get older.

Functional capacity is a tool for assessing frailty [11,12], presenting itself as a vulnerable state where disability, risk of falls, hospitalisation, institutionalisation, and mortality arise [12,13,14,15]. Functional impairment is a growing global problem that increases with age and acute hospitalisations [16]. Older adults who engage in physical exercise have better physiological function than sedentary adults [8,17], and it has even been observed that physical exercise interventions with hospitalised older adults have proven effective in reversing the functional impairment [18] and functional disability [19] associated with hospitalisation [13]. Therefore, we cannot consider age as a problem for significantly improving muscle mass and functional capacity [20,21], since the decrease in strength is due more to a lack of nerve stimulation than to alteration of muscle fibre [22].

The first parameter for detecting probable sarcopenia is a low muscle strength (tools to test this are the “Handgrip strength” or “Chair stand test”). The second criterion used to confirm the diagnosis is to detect a low quantity or quality of muscle, and if the third criterion is met—a low physical performance—it would be considered severe sarcopenia [23]. Strength training is currently the most recommended primary therapeutic strategy to prevent and reverse the decline in muscle mass, strength, and age-related functional impairment [24,25,26]. Strength training also improves the health-related quality of life for older adults at both the mental and physiological levels [27].

HGS is one of the tests used as a predictor of low skeletal muscle strength in the diagnosis of both sarcopenia and frailty [15,28,29,30,31,32], as it correlates with other regions of the body [23].

The aim of this study is to provide reference values of HGS in adults and older adults in the Basque Country by identifying cut-off points to measure weakness and compare the values with other populations. We hypothesize that there are HGS differences in gender and age groups for our sample.

## 2. Materials and Methods

### 2.1. Research Design

This is a descriptive, cross-sectional study, in which the dynamometric and anthropometric measurements were collected from a group of adults and older adults on a health-promoting programme, which were then compared with reference measurements from a similar population in other countries.

### 2.2. Participants

The participants (*n* = 1869) were selected by non-probabilistic convenience sampling from a group of adults and older adults that belong to a municipal programme called “Health for the Elderly”. This programme, despite the use of physical activity (hereinafter, PA) as a vehicle for classes, was oriented towards health and not towards improving sports performance. There was no unified health-enhancing PA programme for all participants, as it was a comprehensive programme in which 12 instructors participated and each performed different activities to meet the programme’s objective of improving the health of the population.

The study was approved by the University of Deusto Ethics Committee (reference # ETK-32/18–19) and written informed consent was obtained from each participant prior to study.

### 2.3. Data Collection

Participants began the programme in October and ended in June. Data collection was done in May by specialized physical activity specialists. Evaluations were conducted at the centres and at the times they normally attended the health-promoting programme, with the evaluator being the one who went to the centres. There were no criteria for inclusion in the participation of the study other than simply being in the database for the programme reported by the City of Bilbao (2868 participants were registered in the database). There were subjects excluded from taking part in the variables extracted by bioimpedance due to the fact that participants with an implanted electronic medical device, or those who have had a stent inserted, should not use this type of platform.

### 2.4. Equipment

Individuals were examined in the centres where they carried out the programme run by the Bilbao City Council to rule out any contraindication to physical exercise.

HGS was obtained using a Camry EH101 electronic hand dynamometer, qualified as medical equipment by the Spanish Agency for Medicines and Health Products. The protocol used was standing with slight shoulder abduction (about 10°), the elbow in full extension, and the forearm and hand in a neutral position [33]. Each person was tested twice and the higher of the two values was obtained. Previous research suggested that different types and brands of dynamometers produce similar results [34].

A Tanita HR 001 Leicester portable stadiometer for height and a Tanita BC-601 Segment (bioimpedance platform) analysis scale for body composition analyses (weight, body fat percentage (hereinafter, % fat), and kilograms of muscle (hereinafter, kg muscle)) were used.

### 2.5. Statistical Analysis

IBM SPSS Statistics software (version 26) was used to conduct the analyses. A two-way ANOVA test was performed with HGS absolute and relative as the dependent variable to analyse differences in the main effects (gender and age groups) and the interaction effects between the age and gender groups. On the other hand, considering the missing values during data collection, a 3 × 2 multivariate ANOVA was conducted for variables such as Body Mass Index (hereinafter, BMI), % fat, and kg muscle to analyse the gender differences, age groups, and the interaction between age and gender groups. In addition, Pearson’s correlations were made between variables such as HGS absolute, HGS relative, BMI, % fat, and kg muscle. A post-hoc analysis was conducted using the least squared difference (LSD) test. Percentiles P5, P10, P25, P50, P75, P90, and P95 were chosen as the age- and sex-specific reference values.

The results obtained for the HGS variable were compared with those for other populations: Great Britain [35], Germany [34], South Korea [36], and Colombia [37]. The effect size was calculated using Cohen’s d to analyse the standardised mean difference (SMD) (mean of changes divided by the difference in means). For this purpose, the standard deviation of N was taken because of the large sample size (except in the Colombian population, where the sample size was used, as the SD was not available); an effect size of 0.2–0.49 was considered small, 0.5–0.79 moderate, and 0.8 or greater as high. Student’s t was obtained with the formula of the sample mean subtracted from the population mean, divided by the population standard deviation where appropriate (for larger size), divided by the square root of the sample. The level of significance was obtained at 95% of the degree of freedom based on the number of sample subjects.

## 3. Results

A total of 1869 subjects who participated in the health-promoting programme for the elderly run by the Bilbao City Council were assessed and the mean values of the variables (HGS, BMI, % fat, and kg muscle) were obtained according to age distribution. Of the 1869 subjects (77.9 years ± 5.7), 87.5% were women (77.8 years ± 5.8) and 12.5% men (78.8 years ± 5.1). When separating by age groups, 7.86% were <70 years old, 18.78% between 70 and 74 years old, 30.18% between 75 and 79 years old, 31.57% between 80 and 84 years old, and 11.61% over 85 years old (Table 1).

Table 2 shows the age- and sex-specific percentiles of HGS in men and women, observing cut-off values for detection of weak HGS with <1 SD by sex and age group. Cut-off points varied from 31.09 to 23.85 and from 18.96 to 12.42 in men and women (Table 2).

The means of the HGS variables (absolute (handgrip kg) and relative (handgrip kg/weight kg)), BMI, % fat, and kg muscle, together with the analysis of differences between age groups, are shown in Table 3. In the analysis of the HGS variable, four women aged 55–59 years and one man > 95 years were removed because of the low volume of the sample. For the analysis of the other variables (BMI, % fat, and kg muscle), 1402 participants were reported with data on all four variables (the loss of subjects is due to the fact that participants with an implanted electronic medical device, or those who had a stent placed, should not use the bioimpedance platform). Thus, the performance of Pearson’s correlation showed statistically significant data between HGS absolute and HGS relative (r = 0.815, *p* < 0.001); HGS absolute and % fat (r = −0.253, *p* < 0.001); HGS absolute and kg muscle (r = 0.562, *p* < 0.001); BMI and % fat (r = 0.668, *p* < 0.001); and BMI and kg muscle (r = 0.428, *p* < 0.001). Although, HGS absolute versus % fat and BMI versus kg muscle gave us low Pearson’s *p* values, which still have statistical significance due to the size of the sample. HGS absolute versus BMI (r = 0.025, *p* = 0.341) did not give a significant correlation. Regarding the HGS relative values, the Pearson’s correlation showed statistically significant results between HGS relative and % fat (r = −0.501, *p* < 0.001); HGS relative and kg muscle (r = 0.127, *p* < 0.001); and HGS relative and BMI (r = −0.418, *p* < 0.001).

**Figure 1:**
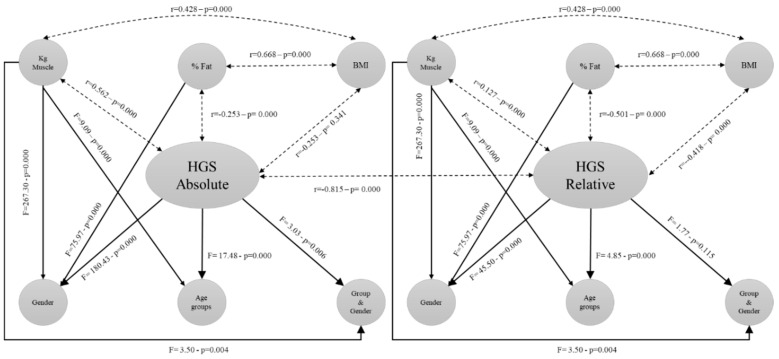
Correlations and mean differences with the absolute and relative handgrip strength (HGS).

The results of the unifactorial ANOVA between the HGS absolute variable between gender (F = 180.43, *p* < 0.001), age groups (F = 17.48, *p* < 0.001), and the interaction of age groups and gender (F = 3.03, *p* = 0.006) show that there are statistically significant differences. The post-hoc analysis indicates that all age groups, except for the 1–3 bands, differ from each other. The HGS relative variable between gender (F = 45.50, *p* < 0.001) and age groups (F = 4.85, *p* < 0.001) show that there are statistically significant differences; on the contrary, the interaction of age groups and gender (F = 1.77, *p* = 0.115) has not shown significant differences. On the other hand, the results of the multifactorial ANOVA reveal that there is a statistically significant difference between the means of gender and % fat (F = 75.97, *p* < 0.001) and kg muscle (F = 267.30, *p* < 0.001), in addition to between the age groups and kg muscle (F = 9.09, *p* < 0.001) and the interaction between age groups and gender with kg muscle (F = 3.50, *p* = 0.004) (Figure 1).

The Table 4 shows the HGS values adjusted by BMI and gender with specific cut-offs and weakness criteria suggested by Fried et al. (2001) [15]. The weakness criterion is defined as the lowest 20% HGS values.

All the results of the dynamometry in different populations (*n* subjects, means and SD, Cohen’s d, Student’s t-test, and level of significance) are shown in Table S1. Dodds et al. (2014) [35] obtained normative data for the HGS from 49,964 UK participants and stipulated percentile curves for HGS for ages 4 to 90. Sample (n) and population-weighted (N) means were obtained in the different age (55–59 = 1, 60–64 = 2, 65–69 = 3, 70–74 = 4, 75–79 = 5, 80–84 = 6, 85–89 = 7, and 90–94 = 8) and gender (w = women and m = men) ranges. The means were for 1 = n (w: 24.7) and N (w: 27.5), for 2 = n (w: 23.5) and N (w: 26.5), for 3 = n (w: 22.4; m: 37.4) and N (w: 25.3; m: 42.3), for 4 = n (w: 21.7; m: 34.6) and N (w: 23.5; m: 39.1), for 5 = n (w: 20.1; m: 32.7) and N (w: 21.4; m: 35.6), for 6 = n (w: 19.3; m: 31.9) and N (w: 19.1; m: 32.2), for 7 = n (w: 17.9; m: 29) N (w: 16.6; m: 28.5), and for 8 = n (w: 17.4) and N (w: 14.2). The results obtained in Cohen’s d were the following; for 1 = w (−0.44), for 2 = w (−0.48), for 3 = (w: −0.49; m: −0.53), for 4 = (w: −0.32; m: −0.55), for 5 = (w: −0.24; m: −0.38), for 6 = (w: 0.03; m: −0.04), for 7 = (w: 0.28; m: 0.07), and for 8 = (w: 0.72).

On the other hand, a comparison was made with the results obtained from Yoo et al. (2017) [36]. They tested 4553 adults over 60 years of age in South Korea for HGS. In this case, they obtained sample (n) and population means (N) in adults over 60 years (1), 65 years (2), and 80 years (3) by gender. The means for women were for 1 = n (20.3) and N (21.6), 2 = n (20.8) and N (20.5), and for 3 = n (18.9) and N (16.7). For men they were for 1 = n (34.2) and N (35.3), 2 = n (34.0) and N (33.6), and for 3 = n (31.1) and N (26.9). The results obtained in Cohen’s *d* were the following; for 1 = (w: −0.31; m: −0.18), for 2 = (w: 0.04; m: 0.05), and for 3 = (w: 0.48; m: 0.70).

Regarding the mean values of the HGS obtained in the study by Ramírez-Vélez et al. (2019) [37] in a Colombian population (*n* = 5237), they also obtained sample (n) and population (N) means in people over 60 years old by gender.

The means for women were n (20.3) and N (16.7) and for men n (34.2) and N (26.7). The results obtained in Cohen’s *d* were as follows; for w (0.63) and for m (0.88).

In addition, cut-off points for HGS were analysed to detect or classify the Colombian population as weak. As shown in Table 2, the five-year means obtained for our subjects are well above the values for Colombia in both men and women.

As far as the results with the German population are concerned, the expected results were not achieved in any age and gender group. Previous research shows differences between different European regions, with a trend towards higher HGS values in northern than in southern European countries [38].

## 4. Discussion

Promoting PA for health has become a priority objective for international bodies and the various public health systems. Poor health, disability, and dependence can be consequences of ageing, but the effectiveness of promoting healthy lifestyles, avoiding sedentarism, and increasing the practice of PA in adults and older adults has been demonstrated to avoid these effects.

Using a representative sample of the Basque Country elderly population (95% confidence interval) at the municipal level (Bilbao Council), from adults and older adults participating in a health-promoting programme, this study highlights the normative values for HGS, observing cut-off points for detecting weak people by age and gender. Reference values for HGS by age group have been published in recent years [34,35,36,37,38,39,40,41,42].

**Figure 2:**
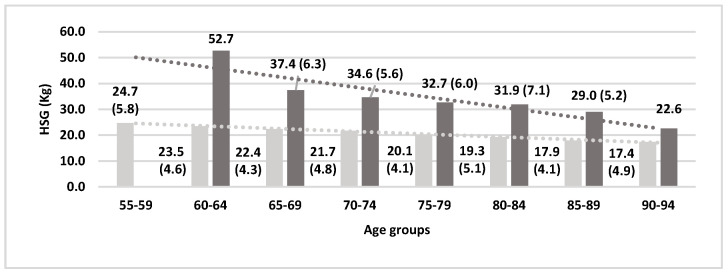
Handgrip strength (HSG) values by five-year age groups.

It was observed that variables such as age and gender were determinants of HGS. The study hypothesis was confirmed with the results. Data obtained from men were significantly (*p* < 0.001) higher than those from women (32.4 kg vs. 20.1), in accordance with numerous studies [34,35,36,37,38,39,40,41,42,43]. Also found to be consistent with other research [26,27,28,29,30,31,32,33,34] was that these values decreased with age: 55–59 = (m: 24.7), for 60–64 = (w: 23.5; m: 52.7), for 65–69 = (w: 22.4; m: 37.4), for 70–74 = (w: 21.7; m: 34.6), for 75–79 = (w: 20.1; m: 32.7), for 80–84 = (w: 19.3; m: 31.9), for 85–89 = (w: 17.9; m: 29), for 90–94 = (w: 17.4), and for ≥95 = (m: 32.8), as we can see in Figure 2.

The data in Table 2 show that men achieved better results in the dynamometric test in all age ranges than women (65–69 = 40.3%, for 70–74 = 37.5%, for 75–79 = 38.3%, for 80–84 = 39.6%, and for 85–89 = 40.3%), observing a sustained trend over time between genders of about 40%. On the other hand, there was a decrease in muscle strength throughout the age range in both genders (w = 26.3%, −6.2 kg and m = 22.4%, −8.4 kg). In addition, the cut-off points for HGS were analysed to detect or classify the Colombian population as weak, and it was observed that the five-year means obtained in our subjects were well above the values for Colombia, in both men and women, in line with the results compared by Ramírez-Vélez et al. (2019) [37] with other more developed populations.

We already confirmed that the higher the age, the lower the HGS. Strength, as can be seen in Table 3, is related to the kg of muscle (r = 0.562), although this decrease may be due to the ageing process itself. The cut-off points for HGS could be used as an indicator of a low amount of muscle mass, being an alternative to identify people likely to undergo a more exhaustive analysis so that the amount of muscle mass can be assessed. They can also contribute to identifying people over 55 years of age who may benefit from modifying their lifestyles to preserve muscle strength, as it can serve as a prediction of hospital admission [44]. Moreover, HGS have been reported in Table 3 as absolute and relative values (HGS kg per weight kg). The statistical analysis of the HGS relative variable showed very similar results to HGS absolute variable. Both variables were strongly correlated (r = 0.815, *p* < 0.001). HGS relative also showed a statistically significant correlation with BMI (r = −0.418, *p* < 0.001), which could be explained by the calculation of BMI that includes the weight of the people tested. On the other hand, HGS relative did not show statistical differences in the interaction between gender and age groups (F = 1.77, *p* = 0.115). These data may contribute to the debate of using either the HGS absolute or relative variable, which McGrath (2019) mentioned in his study [45].

Other authors [15], in their description of the phenotype of fragility, assessed the weakness of HGS in the lowest 20%, adjusted for gender and BMI, as one of the five elements identifying frailty in the population. According to this study, we calculated the weakness points for our sample that could be a good reference for future research studies and could help health professionals assessing weakness in the elderly in this area.

**Figure 3:**
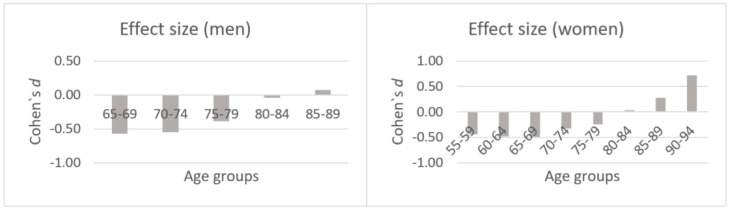
Effect size by five-year age groups in our sample vs. the Great Britain population.

When compared with the British population, it can be observed in Figure 3 that, as age increases, the difference adjusts to the same levels of strength in the HGS test and that the older the person, the better the values of the health-promoting programme, both in men and women. This trend was also observed with the population of South Korea.

Participants in the health-promoting programme were compared as having better average values than the South Korean population over 80 years (w: 0.48 Cohen’s *d* and m: 0.70 Cohen’s *d*) and also better than the Colombian population over 60 years of age; in this case, the size of the effect was moderate for women (0.63) and high for men (0.88).

Therefore, in general, adults over 55 years old in the Basque Country who participate twice a week in a health-promoting programme have better HGS values with respect to other populations as they get older, as it is not clear which factor (genetic, biological, or environmental) is more decisive in obtaining better scores in manual dynamometry [46].

There were no collected comorbidities, muscle pathologies, or the use of certain drugs that can cause interaction in the neuromuscular system. Some limitations to our study included the lack of knowledge of the PA level of our sample and of the compared samples. We know that our sample participates in a health-promoting programme twice a week (100 min per week), in which PA is used to enhance health; however, it is not the only focus of the programme. Therefore, the amount of time spent attending the programme may not be used as the amount of PA time, which also does not meet the minimum PA recommendations set by international organisations [47,48]. Likewise, the PA level of the other samples was also unknown; therefore, in the comparisons, we assume the international samples as the control groups. Another limitation is the lack of a control group, to compare our sample with a group of similar individuals in terms of geographical proximity, genetics, and other variables, which could all influence the result of the HGS test. There are studies that use an international cohort [38] as a control group for comparison [49]; in our study, we observed that our sample is similar to the results obtained by Bohannon et al. [38], in that HGS decreases with age. However, we cannot consider the data from Bohannon et al. [38] as a similar control group.

It should be noted that one of the characteristics of the studied programme is that the average age increased from 72.4 years in 2008 to 77.9 years in 2018, and as age is the only criteria for access to the programme, new participants start out increasingly older and with greater functional disability. This is why favourable results with respect to other populations may only be shown at very advanced ages.

## 5. Conclusions

In an increasingly ageing society like ours, the real challenge is to maintain autonomy and independence as we age, with people’s quality of life being a major challenge.

A health-promoting programme seems to be effective in obtaining better values as age increases with respect to the general population in the HGS test, delaying and even avoiding reaching the cut-off values for detecting weakness as a criterion for frailty.

Despite the current findings available to health professionals for more effective detection of frailty, many of them have not been yet translated into clinical practice. Determining HGS values by population will allow to obtain clinically fast and effective cut-off values to detect weakness and probable risk in an ageing population.

This study provides data on absolute and relative HGS. There are advantages and disadvantages for the use of HGS relative. There is a need for studies that report HGS relative, which could help decide which measure, HGS absolute or relative, has more practical applications, has better use, and could be the standard measure that health professionals use in future detection of frailty.

## Figures and Tables

**Table 1:** Results by age groups and gender for handgrip strength (kg) (*n* = 1869) *.

Age	Subjects		Women		Men	
	Men	Women	Mean	SD	Mean	SD
55–59	0	4	24.7	5.8	-	-
60–64	1	24	23.5	4.6	52.7	-
65–69	9	109	22.4	4.3	37.4	6.3
70–74	35	316	21.7	4.8	34.6	5.6
75–79	76	488	20.1	4.1	32.7	6.0
80–84	82	508	19.3	5.1	31.9	7.1
85–89	28	162	17.9	4.1	29.0	5.2
90–94	1	25	17.4	4.9	22.6	-
95	1	0	-	-	32.8	-
TOTAL	1869		20.1 ^^^	4.7	32.4 ^^^	6.6

* Data presented as the mean (SD) of the dominant hand. (-) There is no SD as there is only one subject. ^^^ Age groups and gender significant differences (*p* < 0.001).

**Table 2:** Age-specific and gender-specific percentiles for handgrip strength (HSG) and the cut-off point values compared with the Colombian population.

Gender/Age Groups	P5	P10	P25	P50	P75	P90	P95	CP *	Mean	SD	CPC	ΔCP
Women (*n* = 1632)												
60–64 (*n* = 24)	17.1	17.9	19.9	22.4	26.7	30.7	32.4	18.9	23.5	4.5	10.1	8.8
65–69 (*n* = 109)	14.8	16.5	19.3	22.0	25.7	28.1	29.7	18.0	22.3	4.3	8.9	9.1
70–74 (*n* = 316)	14.3	16.0	18.4	21.2	24.2	27.4	29.7	16.8	21.6	4.8	8.2	8.6
75–79 (*n* = 488)	13.4	15.5	17.5	20.2	22.5	24.9	26.8	16.0	20.1	4.1	6.7	9.3
80–84 (*n* = 508)	12.5	13.8	16.3	18.9	21.4	24.1	26.0	14.2	19.2	5.0	5.3	8.9
85–89 (*n* = 162)	10.2	13.6	15.5	18.0	20.0	22.5	24.5	13.7	17.9	4.1	4.9	8.8
90–94 (*n* = 25)	9.0	10.4	14.6	18.1	19.2	26.3	28.6	12.4	17.3	4.9		
Men (*n* = 232)												
60–64 (*n* = 1)	-	-	-	-	-	-	-	-	-	-	17.4	
65–69 (*n* = 9)	26.1	26.1	32.0	39.1	43.1	-	-	31.0	37.4	6.3	15.7	15.3
70–74 (*n* = 35)	25.8	26.5	29.9	33.8	37.9	43.3	45.6	28.3	34.6	5.6	14.3	14.0
75–79 (*n* = 76)	21.4	24.2	29.7	33.2	36.0	40.0	43.3	26.6	32.6	6.0	12.3	14.3
80–84 (*n* = 82)	20.4	23.2	27.5	31.8	36.1	39.6	41.1	24.8	31.8	7.0	10.1	14.7
85–89 (*n* = 28)	21.2	22.6	25.7	28.0	32.5	36.6	39.7	23.8	29.0	5.1	8.6	15.2
90–94 (*n* = 1)	-	-	-	-	-	-	-	-	-	-		

* Weak handgrip cut-off point values using <1 SD by gender and age groups. CP = cut-off Point; CPC = cut-off point Colombia; ΔCP = difference between CP − CPC.

**Table 3:** Age groups and gender differences.

			HSG		HSG	BMI	% Fat	kg Muscle
G	Gr	Age Groups	Mean (Absolute)	*n* *	Mean (Relative)			
Women	1	60–64 (*n* = 24)	23.5 (4.5)	21	0.32 (0.08)	29.3 (5.0)	39.0 (5.6)	40.4 (4.4)
	2	65–69 (*n* = 109)	22.3 (4.3)	91	0.32 (0.08)	29.2 (4.9)	39.4 (5.7)	39.5 (4.0)
	3	70–74 (*n* = 316)	21.6 (4.8)	238	0.30 (0.08)	28.9 (4.3)	38.9 (5.9)	39.2 (4.1)
	4	75–79 (*n* = 488)	20.1 (4.1)	346	0.29 (0.07)	28.3 (3.7)	38.4 (5.3)	37.9 (3.8)
	5	80–84 (*n* = 508)	19.2 (5.0)	411	0.28 (0.08)	28.4 (4.1)	38.9 (5.4)	37.6 (3.9)
	6	85–89 (*n* = 162)	17.9 (4.1)	127	0.26 (0.07)	28.1 (4.3)	38.6 (5.6)	37.2 (4.1)
	7	90–94 (*n* = 25)	17.3 (4.9)	21	0.27 (0.06)	27.1 (3.2)	35.6 (4.6)	36.5 (4.9)
		Total	20.0 (4.7)	1255	0.29 (0.08)	28.5 (4.1)	38.7 (5.5)	38.1 (4.0)
Men	1	60–64 (*n* = 1)	52.7	-		-	-	-
	2	65–69 (*n* = 9)	37.4 (6.3)	7	0.45 (0.12)	28.2 (5.0)	29.2 (5.3)	52.6 (6.0)
	3	70–74 (*n* = 35)	34.6 (5.6)	24	0.42 (0.08)	29.2 (2.9)	31.9 (7.2)	52.1 (5.9)
	4	75–79 (*n* = 76)	32.6 (6.0)	44	0.39 (0.09)	28.5 (3.0)	31.4 (6.3)	51.2 (5.7)
	5	80–84 (*n* = 82)	31.8 (7.0)	53	0.41 (0.08)	27.4 (3.4)	29.9 (6.1)	48.1 (5.8)
	6	85–89 (*n* = 28)	29.0 (5.1)	18	0.36 (0.10)	28.3 (3.7)	32.6 (5.6)	47.5 (5.9)
	7	90–94 (*n* = 1)	22.6	1	0.27	26.74	17.80	56.80
		Total	32.4 (6.6)	147	0.40 (0.09)	28.2 (3.3)	30.9 (6.4)	49.9 (6.0)
Total	1	60–64 (*n* = 25)	24.6 (7.3) ^4,5,6,7^	21	0.32 (0.08) ^6,7^	29.2 (5.0)	39.0 (5.5) ^7^	40.4 (4.4) ^6,7^
	2	65–69 (*n* = 118)	23.5 (6.0) ^4,5,6,7^	98	0.33 (0.09) ^4,5,6,7^	29.1 (4.9) ^7^	38.6 (6.2) ^7^	40.4 (5.3) ^4,5,6,7^
	3	70–74 (*n* = 351)	22.9 (6.2) ^4,5,6,7^	262	0.31 (0.09) ^5,6,7^	28.9 (4.2) ^5,7^	38.3 (6.3) ^7^	40.4 (5.6) ^4,5,6,7^
	4	75–79 (*n* = 564)	21.8 (6.1) ^1,2,3,5,6,7^	390	0.30 (0.08) ^2,5,6,7^	28.3 (3.6)	37.6 (5.8) ^7^	39.4 (5.8) ^2,3,5,6,7^
	5	80–84 (*n* = 590)	21.0 (6.9) ^1,2,3,4,6,7^	464	0.29 (0.09) ^2,3,4,6^	28.2 (4,0) ^3^	37.8 (6.2) ^7^	38.8 (5.3) ^2,3,4^
	6	85–89 (*n* = 190)	19.5 (5.8) ^1,2,3,4,5,7^	145	0.28 (0.08) ^1,2,3,4,5^	28.2 (4.2)	37.9 (5.9) ^7^	38.5 (5.5) ^1,2,3,4^
	7	90–94 (*n* = 26)	17.5 (4.9) ^1,2,3,4,5,6^	22	0.27 (0.06) ^1,2,3,4^	27.1 (3.2) ^2,3^	34.8 (5.9) ^1,2,3,4,5,6^	37.5 (6.5) ^1,2,3,4^
		Total	21.6 (6.5)	1402	0.30 (0.09)	28.5 (4.1)	37.9 (6.1)	39.3 (5.6)

Gr = group; G = gender. * Missing values in the measurement of the variables BMI, % fat, and kg of muscle. ^1,2,3,4,5,6,7^ Significant differences < 0.05 between age groups within the total participant sample.

**Table 4:** Handgrip strength, stratified by gender and body mass index (BMI) quartiles.

Men	Cut-off for HGS (kg) Criteria for Frailty	Weakness Criteria *
BMI < 25.6	≤33.6	≤27.8
BMI 25.6–28	≤32.4	≤26.8
BMI 28–30.1	≤33.0	≤29.2
BMI > 30.1	≤32.2	≤26.2
**Women**		
BMI < 25.6	≤19.5	≤16.3
BMI 25.6–28.1	≤19.7	≤15.8
BMI 28.1–31	≤20.3	≤16.6
BMI > 31	≤20.2	≤16.3

* Weakness criterion defined as the lowest 20% HGS values, adjusted for BMI and gender (Fried et al. [15]).

## Supplementary Materials

The following are available online at https://www.mdpi.com/2079-7737/9/12/414/s1, Table S1: Results by age groups and gender in the HGS (kg) in different populations.
