# Supplementary material for: Reference Values for Handgrip Strength in the Basque Country Elderly Population
Source: Biology (Basel). 2020 Nov 24;9(12):414. doi: 10.3390/biology9120414 (PMC7760619; doi:10.3390/biology9120414)
Supplement: Supplementary file 1 [file biology-09-00414-s001.pdf]

Table S1. Results by age groups and gender in the HGS (kg) in different populations.

Gender/ Age	Basque			Great Britain [1]			Germany [2]			South Korea [3]			Colombia [4]			Comparison of averages 1				2				3				4*			
**Women**	*n*	*m*	*SD*	*N*	*m*	*SD*	*N*	*m*	*SD*	*N*	*m*	*SD*	*N*	*m*	*SD*	*d*	*t*	$t_{95\%.df}$£	*sig.*	*d*	*t*	$t_{95\%.df}$£	*sig.*	*d*	*t*	$t_{95\%.df}$£	*sig.*	*d*	*t*	$t_{95\%.df}$£	*sig.*
55-59	4	24.7	5.8	3743	27.5	6.4	1092	30.0	5.5	275	24.3	4.1	-	-	-	-0.44	-0.88	3.18	no	-0.97	-1.94	3.18	no	0.10	0.20	3.18	no	-	-	-	-
60-64	24	23.5	4.6	2683	26.5	6.2	1004	29.0	5.3	221	23.8	3.9	873	18.5	-	-0.48	-2.36	2.06	yes	-1.04	-5.07	2.06	yes	-0.08	-0.39	2.06	no	1.10	5.39	2.06	yes
65-69	109	22.4	4.3	3947	25.3	6	947	27.6	5.2	184	22.8	4.6	774	17.3	-	-0.49	-5.12	1.97	yes	-1.01	-10.52	1.97	yes	-0.09	-0.91	1.97	no	1.17	12.26	1.97	yes
70-74	316	21.7	4.8	3286	23.5	5.7	895	26.0	4.9	145	21.1	4.5	563	16.1	-	-0.32	-5.74	1.96	yes	-0.89	-15.74	1.96	yes	0.12	2.15	1.96	yes	1.15	20.42	1.96	yes
75-79	488	20.1	4.1	1883	21.4	5.4	522	24.2	4.5	107	18.9	4.2	427	14.7	-	-0.24	-5.32	1.96	yes	-0.91	-20.13	1.96	yes	0.29	6.37	1.96	yes	1.31	29.02	1.96	yes
80-84	508	19.3	5.1	1115	19.1	5.1	-	-	-	-	-	-	271	13.4	-	0.03	0.75	1.96	no	-	-	-	-	-	-	-	-	1.16	26.20	1.96	yes
85-89	162	17.9	4.1	1134	16.6	4.7	-	-	-	-	-	-	-	-	-	0.28	3.60	1.97	yes	-	-	-	-	-	-	-	-	-	-	-	-
90-94	25	17.4	4.9	431	14.2	4.4	-	-	-	-	-	-	-	-	-	0.72	3.58	2.06	yes	-	-	-	-	-	-	-	-	-	-	-	-
80-90	670	18.6	4.6	2249	17.9	4.9	420	21.4	4.1	-	-	-	-	-	-	0.15	3.96	1.96	yes	-0.68	-17.68	1.96	yes	-	-	-	-	-	-	-	-
>60	1632	20.3	4.6	14479	20.9	5.4	3788	25.6	4.8	848	20.7	4.3	3065	16.7	5.7	-0.12	-4.85	1.96	yes	-1.11	-44.94	1.96	yes	-0.08	-3.38	1.96	yes	0.63	31.62	1.98	yes
>65	1608	20.8	4.5	11796	20.0	5.2	2784	24.8	4.7	627	19.9	4.4	-	-	-	0.14	5.66	1.96	yes	-0.87	-34.72	1.96	yes	0.19	7.77	1.96	yes	-	-	-	-
>80	695	18.9	4.9	2680	16.6	4.7	420	21.4	4.1	191	16.7	4.5	-	-	-	0.48	12.57	1.96	yes	-0.61	-16.14	1.96	yes	0.48	12.68	1.96	yes	-	-	-	-
**Men**																															
55-59	-	-	-	4250	46	9.8	1010	49.1	8.5	234	40.2	6.1	-	-	-	-	-	-	-	-	-	-	-	-	-	-	-	-	-	-	-
60-64	1	52.7	-	2943	45	9.2	950	46.3	8.4	165	38.7	6.6	562	30.5	-	-	-	-	-	-	-	-	-	-	-	-	-	-	-	-	-
65-69	9	37.4	6.3	4171	42	8.6	1019	44.1	7.3	165	36.9	5.6	535	28.6	-	-0.57	-1.70	2.30	no	-0.92	-2.75	2.30	yes	0.09	0.28	2.30	no	1.39	4.18	2.30	yes
70-74	35	34.6	5.6	3473	39	8.1	915	41.7	7.6	102	33.7	5.4	423	26.3	-	-0.55	-3.26	2.02	yes	-0.93	-5.50	2.03	yes	0.17	1.01	2.03	no	1.49	8.80	2.03	yes
75-79	76	32.7	6.0	2135	36	7.6	582	37.8	7.9	92	31.8	6.6	304	23.8	-	-0.38	-3.35	1.99	yes	-0.65	-5.65	1.99	yes	0.14	1.22	1.99	no	1.48	12.86	1.99	yes
80-84	82	31.9	7.1	1361	32	7.3	-	-	-	-	-	-	201	20.4	-	-0.04	-0.40	1.98	no	-	-	-	-	-	-	-	-	1.62	14.68	1.98	yes
85-89	28	29.0	5.2	1632	29	7	-	-	-	-	-	-	-	-	-	0.07	0.38	2.05	no	-	-	-	-	-	-	-	-	-	-	-	-
90-94	1	22.6	-	702	25	6.8	-	-	-	-	-	-	-	-	-	-	-	-	-	-	-	-	-	-	-	-	-	-	-	-	-
80-90	110	30.4	6.1	2993	30.4	7.2	350	33.2	7.5	-	-	-	-	-	-	0.01	0.13	1.97	no	-0.37	-3.86	1.97	yes	-	-	-	-	-	-	-	-
>60	232	34.2	6.6	16417	35.3	7.8	3816	40.6	7.7	524	35.3	6.0	2172	26.7	8.5	-0.14	-2.12	1.96	yes	-0.83	-12.63	1.96	yes	-0.18	-2.68	1.96	yes	0.88	13.44	1.96	yes
>65	231	34.0	6.2	13474	33.7	7.6	2866	39.2	7.6	415	32.3	5.9	-	-	-	0.03	0.45	1.96	no	-0.69	-10.52	1.96	yes	0.28	4.27	1.96	yes	-	-	-	-
>80	111	31.1	6.7	3695	28.5	7.0	350	33.2	7.5	56	26.9	6.0	-	-	-	0.37	3.93	1.97	yes	-0.28	-2.96	1.97	yes	0.70	7.39	1.97	yes	-	-	-	-

HGS=handgrip strength. *n*=number of sample subjects. *N*=number of population subjects. *m*= Mean HGS (kg). *SD*=Standar Desviation.

[1] Obtained from the study of Doods et al., 2014 - [2] Obtained from the study of Steiber. N. 2016. - [3] Obtained from the study of Yoo et al., 2017. - [4] Obtained from the study of Ramirez Velez et al., 2019.

£ The critical value of t at 5%, depending on the sample size in each case.

* For the calculation of Cohen's *d* and Student's *t* we used the *SD* of the sample because we do not have *SD* (except in >60 years) in study of Ramírez Velez et al.. 2019.
